# Stem Cells in the Myelodysplastic Syndromes

**DOI:** 10.3389/fragi.2021.719010

**Published:** 2021-07-16

**Authors:** Di Zhan, Christopher Y. Park

**Affiliations:** ^1^ Department of Pathology, New York University Grossman School of Medicine, New York, NY, United States; ^2^ Perlmutter Cancer Center, New York University Grossman School of Medicine, New York, NY, United States

**Keywords:** myelodysplastic syndromes, hematopoietic stem cells, novel therapeutics, clonal hematopoiesis, acute myeloid leukemia

## Abstract

The myelodysplastic syndromes (MDS) represent a group of clonal disorders characterized by ineffective hematopoiesis, resulting in peripheral cytopenias and frequent transformation to acute myeloid leukemia (AML). We and others have demonstrated that MDS arises in, and is propagated by malignant stem cells (MDS-SCs), that arise due to the sequential acquisition of genetic and epigenetic alterations in normal hematopoietic stem cells (HSCs). This review focuses on recent advancements in the cellular and molecular characterization of MDS-SCs, as well as their role in mediating MDS clinical outcomes. In addition to discussing the cell surface proteins aberrantly upregulated on MDS-SCs that have allowed the identification and prospective isolation of MDS-SCs, we will discuss the recurrent cytogenetic abnormalities and genetic mutations present in MDS-SCs and their roles in initiating disease, including recent studies demonstrating patterns of clonal evolution and disease progression from pre-malignant HSCs to MDS-SCs. We also will discuss the pathways that have been described as drivers or promoters of disease, including hyperactivated innate immune signaling, and how the identification of these alterations in MDS-SC have led to investigations of novel therapeutic strategies to treat MDS. It is important to note that despite our increasing understanding of the pathogenesis of MDS, the molecular mechanisms that drive responses to therapy remain poorly understood, especially the mechanisms that underlie and distinguish hematologic improvement from reductions in blast burden. Ultimately, such distinctions will be required in order to determine the shared and/or unique molecular mechanisms that drive ineffective hematopoiesis, MDS-SC maintenance, and leukemic transformation.

## Introduction

The myelodysplastic syndromes (MDS) are a group of clonal myeloid disorder characterized by ineffective hematopoiesis, disordered hematopoiesis evidenced by morphologic dysplasia, and frequent progression to acute myeloid leukemia (AML). Chemotherapy, stem cell transplantation, hypomethylating agents (HMAs) including decitabine (DAC) and azacytidine (AZA), and immunomodulatory drugs such as lenalidomide are used to treat MDS patients (Platzbecker, 2019). Although these treatment strategies can induce clinical remissions accompanied by improvement in hematologic parameters, all patients will eventually become refractory, and relapse occurs in all MDS patients in the absence of curative therapy, which is currently limited to allogeneic transplantation. It has been proposed that MDS is a disease of stem cells and that MDS-SCs persist during therapy and expanded at the time of relapse. The evolution of stem cell clones from the pre-malignant to malignant state is recognized to play a critical role in MDS pathogenesis and disease progression. This review will summarize the alterations in surface proteins and signaling pathways in MDS-SCs, as well as clonal evolution patterns in MDS-SCs and emerging therapies that target them.

## Hematopoietic Stem and Progenitor Cell Alterations in MDS

**FIGURE 1:**
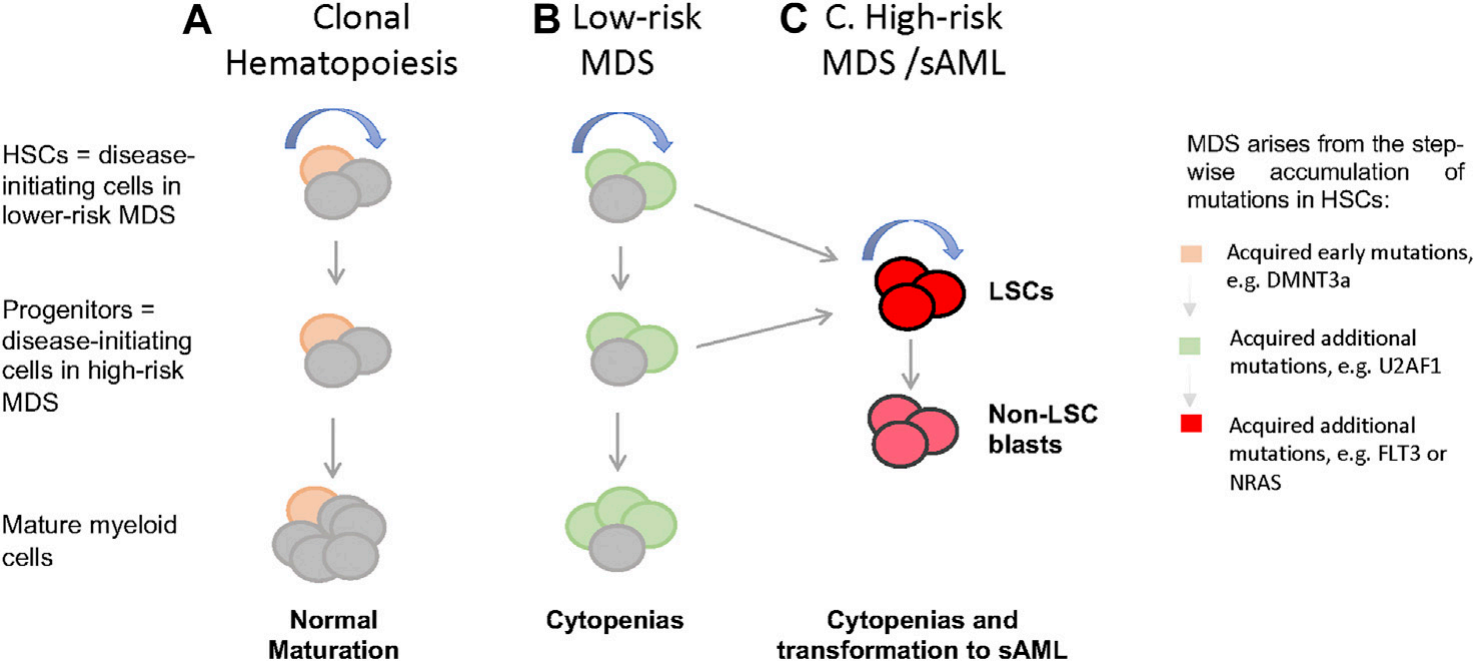
Cellular and genetic hierarchies and disease progression in MDS. **(A)** In healthy individuals, hemotopoiesis is charcterzied as a hierarchy in which self-renewing HSCs give rise to non-self-renewal multipotent and commited progintors and eventually mature cells. When aged HSCs acquire somatic mutations and give rise clones with enhanced self-renewal (yellow), thsi is detectable in the peripheral blood as clonal hematopoiesis (CH), which often characterized by mutations in genes such as DMNT3a, TET, and ASXL1. **(B)** when HSCs harboring CH associated mutations acquire additional mutations in genes such as U2AF1, SRF2, and SF3B1, this leads to the formation of MSD stem cells, MDS-SCs (green). In low-risk MDS, disease stem cells immunophenotypically resemble normal HSCs, while in high-risk disease with excess blasts, resmbles committed myeloid progenitors. **(C)** It is presumed that acquisitions of lesions such as FLT3 and NRAS mutations in HSCs or committed proginitors drive the formation of leukemia stem cells (LSCs) and non-self-renewing blasts that accumalate in high-risk MDS and sAML. LSCs immunophenotypically resemble committed progenitors. (curved blue arrow = self-renewal).

MDS can be classified into different clinical risk categories based on hematopoietic features, age, and cytogenetic/genetic information at diagnosis. The International Prognostic Scoring System (IPSS) divides MDS into low-risk (Low and Intermediate-1 [Int-1]) and high-risk (Int-2 and High) disease, with high-risk MDS associated with higher blast counts, increased incidence of leukemic transformation, and poor clinical outcome (Greenberg et al., 2012; Greenberg et al., 1997). Studies of low-risk MDS showed no increase in the frequency of immunophenotypically defined HSCs (Lin-CD34+CD38-CD90+CD45RA-) (Majeti et al., 2007; Pang et al., 2013), although multiple studies observed loss of granulocyte-macrophage progenitors (GMPs, Lin−CD34+CD38+CD123+CD45RA+) and relative expansion of common myeloid progenitors (CMPs, Lin−CD34+CD38+CD123+CD45RA−) in the bone marrow (BM) of low-risk MDS patients. However, in high-risk MDS [i.e., refractory anemia with excess blasts (RAEB)], GMP frequency was increased compared to healthy individuals (Pang et al., 2013; Will et al., 2012). These studies underscore that MDS with lower and higher blast counts likely represent distinct biologic entities characterized by unique alterations in HSPC number and frequency (Figure 1). Intriguingly, Will et al., showed that phenotypically primitive long-term HSCs (LIN-CD34+CD38-CD90+) in MDS are expanded in higher-risk cases, suggesting that alterations in HSC function are required prior to the accumulation of blasts (Figure 1; Will et al., 2012). Reduction of megakaryocyte-erythroid progenitors (MEPs, Lin−CD34+CD38+CD123-CD45RA−) can be observed on both high-risk and low-risk MDS, with a relatively greater reduction in low-risk MDS (Will et al., 2012; Pang et al., 2013), suggesting a differentiation block in the transition from CMPs to MEPs.

## MDS is a Hematopoietic Stem Cell Disease

The cancer stem cell hypothesis holds that cancers are initiated and propagated by a rare stem cell population that has the unique ability to self-renew and replenish non-self-renewing malignant cells. Multiple studies have shown that leukemia stem cells (LSCs) in AML immunophenotypically resemble committed progenitor cells such as lymphoid-primed multi-potent progenitors (LMPPs) (Lin-CD34+CD38-CD90-CD45RA+) or GMPs (CD34+CD38+CD123+/loCD110-CD45RA+) (Krivtsov et al., 2006; Goardon et al., 2011; Shlush et al., 2017; van Galen et al., 2019). MDS, in particular lower-risk MDS cases without excess blasts, has been shown to originate in neoplastic HSCs. Earlier studies showed that MDS reconstituting activity is exclusively derived from the CD34+CD38-CD90+ HSC population (Nilsson et al., 2002), demonstrating that MDS initiating cells have a similar phenotype as normal HSCs. Tehranchi et al. evaluated hematopoietic stem and progenitor cell (HSPC) populations in del (5q) MDS patients treated with lenalidomide in remission and relapse (Tehranchi et al., 2010) and identified rare and phenotypically distinct del (5q) MDS-SCs (CD34+CD38−/low CD90+) that are resistant to lenalidomide treatment (Tehranchi et al., 2010). The first xenograft studies demonstrating that HSCs are functional disease-initiating cells in MDS were performed by Pang et al., when they transplanted purified HSCs (LIN-CD34+CD38-CD45RA-CD90+) from MDS samples into immunodeficient mice (Pang et al., 2013). Subsequently Woll et al. demonstrated that HSCs (LIN- CD34+CD38-CD45RA-CD90+), but not CMP, GMP, or MEP, are able to generate long-term cell cultures *in vitro* and maintain long-term engraftment in immunodeficient mice. Taken together, these studies demonstrate that MDS is a disease that is maintained and propagated by HSCs. In more recent studies exploring the role of the hematopoietic niche in MDS, studies have shown that HSPCs from low-risk MDS patients can reprogram healthy mesenchymal stromal cells (MSCs), and that the reprogrammed MSCs promote MDS HSC engraftment in xenografts (Medyouf et al., 2014). In addition, MDS-SCs from cases with and without excess blasts have been shown to preferentially migrate and engraft into human mesenchymal cell-seeded scaffolds in immunodeficient mice, suggesting that mesenchymal cells interact with MDS-SCs and provide a niche that supports MDS-SCs self-renewal (Mian et al., 2021).

## Dysregulated Signaling Pathways in MDS HSCs

Positive regulators of inflammation and innate immunity have been shown to play an important role in promoting MDS pathogenesis. Activation of the p38 MAPK, tumor necrosis factor alpha (TNF-a), and transforming growth factor beta (TGF-b) pathways can promote the expansion of normal HSPC populations while increasing apoptosis in more mature populations (Allampallam et al., 2002; Verma et al., 2002; Saberwal et al., 2003; Navas et al., 2008). HSCs also can be directly activated by pathogen recognition receptor like TLRs or proinflammatory cytokines. Studies have shown that TLRs 1, 2, and 6 are overexpressed in MDS HSPCs (Wei et al., 2013). TLR1 and TLR6 bind to TLR to activate nuclear factor kB (NF-kB), p38 MAPK, and interleukin-1 receptor-associated kinase 1 (IRAK1) signaling pathways in MDS HSPCs (Wei et al., 2013). Overstimulation of these pathways leads to MDS HSPC expansion and a block in erythroid differentiation (Wei et al., 2013). Recent studies also showed IRAK1 is significantly overexpressed in MDS CD34+ cells compared to cord blood CD34+ cells and that IRAK1 phosphorylation leads to TRAF6 and downstream NF-kB activation in MDS HSPCs in both low- and high-risk disease (Rhyasen et al., 2013). In addition, transgenic TRAF6 overexpression in mouse HSPCs leads to an MDS-like phenotype *in vivo* (Fang et al., 2017; Muto et al., 2020). A recent study showed that the long isoform of interleukin-1 receptor-associated kinase 4 (IRAK4) was the dominant alternatively spliced isoform and highly expressed in CD34+ HSPCs from MDS and AML patients harboring U2AF1 mutations, and inhibition of the long isoform of IRAK4 abrogated disease growth in AML xenografts. These studies indicate that the long isoform of IRAK4 plays a critical role in the activation of chronic innate immune signaling in MDS and AML and thus is a candidate therapeutic target (Smith et al., 2019).

Several other innate immune signaling pathways have been implicated in MDS. The innate immune cytokine IL-8 and its receptor CXCR2 were found to be concomitantly overexpressed in high-risk MDS-SCs and AML LSCs when compared with healthy counterparts, suggesting that an IL-8/CXCR2 autocrine loop promotes MDS-SC and AML LSC function (Schinke et al., 2015). CXCR2 inhibition or downregulation decreased oncogenic MAPK signaling and lead to cell cycle arrest in MDS and AML CD34+/CD38-cells (Schinke et al., 2015). IL1RAP also has been shown to be upregulated in MDS-SCs and AML LSCs and associated with poor clinical outcomes in high-risk MDS and AML (Barreyro et al., 2012). Increased expression of danger-associated molecular pattern (DAMP) molecules S100A8/S100A9 in MDS patients perpetuates noninfectious inflammatory responses that result in the accumulation of Lin- HLA-DR- CD33+ myeloid-derived suppressor cells (MDSCs) in the BM (Chen et al., 2013; Cluzeau et al., 2017; Cheng et al., 2019). Furthermore, S100A9 binds to CD33, which leads to the expansion of MDSCs and suppression of erythroid and myeloid progenitors in a mouse model of MDS (Chen et al., 2013). S100A9 can induce the upregulation of PD-1/PD-L1 expression in MDSCs from S100A9 transgenic mice, and PD-1/PD-L1 blockade restores effective hematopoiesis and improves colony-forming capacity in BM mononuclear cells (MNCs) from MDS patients (Cheng et al., 2019). Collectively, these studies demonstrate the critical role of innate inflammatory pathways in promoting the survival and expansion of MDS-SCs.

One dysregulated non-inflammatory pathway thought to promote MDS HSCs self-renewal is the immunoglobulin-like and endothelial growth factor-like domains 1 (Tie2) pathway. Angiopoietin 1 (ANGPT1) promotes vascular development and angiogenesis by binding to the receptor tyrosine kinase TIE2, which supports normal HSC quiescence and self-renewal (Arai et al., 2004). Recent studies demonstrated that ANGPT-1 is overexpressed in MDS HSPCs and that high ANGPT1 expression is associated with worse clinical outcomes in high-risk MDS and AML patients (Cheng et al., 2011). In addition, Tie2 knockdown and inhibition suppresses leukemic proliferation and enhances hematopoietic differentiation of patient MDS HSPCs (Bachegowda et al., 2016). Another pathway overactivated in MDS HSPCs is the HIF1a signaling pathway. HIF1a protein expression is significantly upregulated in BM MNCs in a subset of MDS patient specimens and high HIF1a expression is associated with worse outcomes in MDS (Tong et al., 2012). Hayashi et al. showed that HIF1a overactivation leads to a MDS-like phenotype in a mouse transgenic model and that HIF1a activation is essential for HSPC expansion in a MLL-PTD mutant mouse model that presents with MDS-associated features (Hayashi et al., 2018).

## Defective Ribosome Biogenesis in MDS HSPCs

Defective ribosome biogenesis has been implicated in MDS pathogenesis, as loss of RPS14 expression contributes to del (5q) MDS (Ebert et al., 2008). Similarly, diseases of hematopoiesis resulting in decreased ribosomal protein expression or ribosome assembly – so-called “ribosomopathies” – such as Diamond Blackfan anemia show reductions in mature red cell output, similar to MDS (Ebert et al., 2005; Flygare et al., 2005). Indeed, genetic mouse models deficient in other ribosomal proteins including RPS14, RPS19, and RPS6 exhibited reductions in red cell production (Barlow et al., 2010; Jaako et al., 2011; McGowan et al., 2011). These studies from haploinsufficient genetic mouse models of ribosomal proteins also showed the red cell hypoplasia was dependent on activation of P53 (Barlow et al., 2010; Jaako et al., 2011; McGowan et al., 2011). Taken together, these studies demonstrate that defective ribosome biogenesis likely contributes to the cytopenias observed in low-risk MDS.

Although genetic loss of ribosomal proteins can directly contribute to decreased ribosome formation and MDS-like phenotypes, reduced ribosomal protein gene transcripts have been shown to be common in low-risk MDS. Reduced RPS14 transcripts were described in CD34+ HSPCs from MDS patients without del (5q), defining a subgroup of patients with prolonged survival (Czibere et al., 2009). Other studies showed increased ribosomal protein transcript levels in in CD34+ HSPCs from MDS patients (Sridhar et al., 2009). However, when highly purified MDS HSCs were assessed, global reductions in 21 ribosomal genes were identified, even in patients lacking del (5q) (McGowan et al., 2011), suggesting that ribosomal proteins - and thereby global translation – is reduced in MDS HSCs. We speculate that given the more recent findings that HSCs depend on highly regulated (and low) rates of translation (Signer et al., 2014), decreases in global translation and reduced proteotoxic stress may provide a survival benefit to mutant ribosomal protein HSCs in a cell-intrinsic manner, resulting in their selective advantage.

## Genetic Alterations and Clonal Evolution in MDS HSCs

### Cytogenetic Abnormalities in MDS HSPCs

Studies utilizing fluorescence in-situ hybridization (FISH) techniques demonstrated the presence of cytogenetic abnormalities in sorted HSPCs from MDS patients (Nilsson et al., 2007; Tehranchi et al., 2010). Studies of del (5q) MDS patients at the time of diagnosis found that the vast majority of CD34+/CD38+ and CD34+/CD38- HSPCs harbor del (5q). This lesion persisted in CD34+/CD38-/CD90+ MDS HSCs despite continuous lenalidomide treatment, consistent with MDS HSCs being relative resistant to therapy and the source of disease maintenance/re-emergence following therapy (Nilsson et al., 2007; Tehranchi et al., 2010). Other studies confirmed that nearly all MDS HSCs harbor cytogenetic alterations such as deletion of chromosome 7 (Will et al., 2012; Pang et al., 2013). Alterations such as trisomy 8, loss of chromosome 7, or deletion of the long arm of chromosome 20 (20q-) also are enriched in MDS HSCs or myeloid progenitors compared to total BM cells or other mature populations such as B-, T- and NK-cells (Miura et al., 2000; Nilsson et al., 2002). Cytogenetically abnormal HSCs also persist through AZA treatment despite achievement of morphologic remission (Will et al., 2012). Despite the fact that many of these cytogenetic abnormalities are considered diagnostic of MDS, these events may not always be “founder” events. For example, Mossner et al. demonstrated that del (5q) is a potential “founder” event in only a minority cases (21.4%) and that cytogenetic lesions including monosomy 7, trisomy 8, and del (5q) can be acquired as late events in MDS (Mossner et al., 2016).

### Driver Mutations in MDS HSPCs

A number of large studies have evaluated the presence of somatic mutations in MDS using whole genome- or targeted exome sequencing. Collectively, these studies have demonstrated that MDS shares many driver mutations with other myeloid neoplasms including AML. Genome-wide sequencing analysis of a large cohort of MDS patients showed a mean number of nonsynonymous mutations of approximately 11 per patient (or 0.34/Mb) for all MDS samples with a lower mutation rate in low-risk MDS (0.19/Mb) (Kim et al., 2017). Using targeted sequencing and mass spectrometry–based genotyping, a study of 439 MDS patient BM aspirates demonstrated that 51% of patients had at least one point mutation present at a mutation allele frequency (MAF) of 10% and above, and that mutations in TP53, EZH2, ETV6, RUNX1, and ASXL1 are predictors of poor overall survival in patients, independent of established risk factors such as age, sex and IPSS risk group (Bejar et al., 2011). Papaemmanuil et al. performed targeted sequencing of 111 genes in 738 patients with MDS or closely related neoplasms [e.g., chronic myelomonocytic leukemia (CMML) and mixed MDS-myeloproliferative neoplasms (MPN)] and found that 78% of cases harbored one or more recurrent driver mutations at a MAF ≥10% (Papaemmanuil et al., 2013). Among many categories of somatic mutations, they found that mutations involving RNA spliceosome components including SF3B1, SRSF2, U2AF1 and ZRSR2 occurred most frequently in MDS and were associated with distinct clinical features (Papaemmanuil et al., 2013). More recent studies assessing exome mutations in a cohort of 2,250 MDS patients identified somatic mutations in 10 genes enriched in high-risk MDS, including GATA2, NRAS, KRAS, IDH2, TP53, RUNX1, STAG2, ASXL1, ZRSR2, and TET2, while SF3B1 mutations were almost exclusively found in lower-risk MDS (Makishima et al., 2017). For additional discussion of the mutational spectrum in MDS, we refer the reader to excellent reviews on this subject (Sperling et al., 2017; Ogawa, 2019).

These genetic studies revealed the prevalence and clinical significance of recurrent driver mutations in MDS. Multiple groups have shown that many of the hematologic features of MDS can be partial recapitulated in single-gene genetic mouse models, such as SRSF2, U2AF, SF3B1, and ASXL1 knock-in or knock-out mouse models (Abdel-Wahab et al., 2013; Kim et al., 2015; Shirai et al., 2015; Mupo et al., 2017). However, many of these mouse models require transplantation to elicit physiologically relevant cytopenias, or to generate cytopenias quickly, raising questions about the importance of the microenvironment as well as whether or not any single gene mutation model can truly model MDS.

### MDS HSCs and Clonal Evolution

Delineating the hierarchical organization of clones in MDS HSCs is critical to understand mechanisms of disease progression and responses to drug therapy in MDS. Clonal structures and mutation hierarchy are inferred using MAF data, assuming mutations with the highest MAFs are clonal and likely occur early, while mutations with lower MAFs are subclonal and likely occur late in disease pathogenesis. Among different categories of oncogenic mutations, alterations in genes involving RNA splicing components including SF3B1, ZRSF2, SRSF2, and U2AF1 are predicted to represent earlier events that dictate disease evolution and are associated with distinct clinical phenotypes (Papaemmanuil et al., 2013). Another sequencing study found mutations in genes related to DNA methylation and splicing machinery occur earlier during disease evolution while mutations related to signaling pathways expand significantly during progression to secondary AML (sAML) (Kim et al., 2017). Using targeting sequencing of patient and xenografted cells, Mossner et al. evaluated clonal heterogeneity and reconstructed mutational trajectories in BM samples from 54 MDS and CMML patients, including 22 patients covering a cumulative observation time of 75 years (Mossner et al., 2016). They found that mutations in epigenetic modifiers including TET2 and ASXL1, and RNA splicing factors including SF3B1 and SRSF2, are the predominant founder events in MDS, while genes involved in signaling cascades including JAK2 and CBL, transcription factors including RUNX1 and ETV6, and cytogenetic lesions including monosomy 7, trisomy 8, and del (5q), were almost exclusively acquired as late events (Mossner et al., 2016). Other studies showed similar findings (Papaemmanuil et al., 2013; Kim et al., 2017; Makishima et al., 2017). While these studies helped identify the order of mutation acquisition during MDS pathogenesis, they did not resolve which mutation or set of mutations is minimally required for the development of MDS. It is worth noting that mutations in ASXL1, TET2, and DNMT3A, but not RNA spliceosome components, are common in hematopoietic cells from healthy elderly individuals exhibiting clonal hematopoiesis (Genovese et al., 2014; Jaiswal et al., 2014; Xie et al., 2014). It is thus thought that alterations in splicing are required for full manifestation of MDS clinical phenotypes.

Newer sequencing approaches now allow concomitant evaluation of genetic alterations and transcriptomes at the single cell level. Chen et al. performed deep targeted sequencing combined with single-cell sequencing on phenotypically defined malignant stem cells (MDS-SC, AML-SC), premalignant stem cells (pre-MDS-SC, pre-AML-SC), and blast populations in 7 patients with MDS who later progressed to sAML (Chen et al., 2019). They found significantly higher subclonal diversity at the MDS-SC level than in blasts in patients with MDS and sAML. Furthermore, they observed that sAML often developed from a rare subclone contained within the (pre-)MDS-SC pool, and not through further evolution of MDS blasts, indicating a parallel, rather than linear, clonal evolution pattern during MDS progression to sAML (Chen et al., 2019). Thus, this study provided novel insights into MDS disease that were previously unrecognized using bulk-cell sequencing approaches. Unfortunately, these studies did not evaluate genetic evolution or diversity in MDS patients with progressive cytopenias or during treatment.

## Therapeutic Targeting of MDS HSCs

Since MDS-SCs are critical for the initiation and propagation of MDS and are the presumed cell that undergoes additional genetic/epigenetic changes to mediate disease progression and relapse, they must be eradicated in order to cure the disease. Not surprisingly, much of the effort to identify therapeutic targets in MDS have focused on high-risk MDS/AML, with relatively few studies directly assessing effects on MDS HSCs in lower-risk disease. This distinction is important to keep in mind when interpreting studies and the potential utility of novel therapeutic strategies in lower-risk MDS. Ideally, therapies in MDS would allow targeting of malignant HSCs without affecting normal HSCs or hematopoiesis. A number of targets have been identified as potential therapeutic targets in MDS.

Multiple studies demonstrated that current therapies including lenalidomide and HMAs treatment are insufficient to eradicate MDS-SCs. Lenalidomide can induce clinical and cytogenetic remissions in MDS patients with del (5q). However, all patients will eventually relapse. A study examined HSPCs from BM specimens obtained from seven MDS patients with del (5q) treated with lenalidomide showed persistence of HSC harboring del (5q), even in patients who achieved cytogenetic remissions and hematologic responses (Tehranchi et al., 2010). AZA treatment leads to increased overall survival in high-risk MDS and was recently been approved for maintenance therapy in AML (Kantarjian et al., 2006; Fenaux et al., 2009; Dombret et al., 2015; Jabbour et al., 2017; Wei et al., 2020). However, responses are difficult to predict, and treatment failure invariably occurs due to the inability of AZA to eliminate MDS-SCs. This was formally demonstrated in a study evaluating LMPP-like and GMP-like populations from 79 patients with high-risk MDS and AML treated with AZA and found that malignant stem cells were never completely eradicated and that expansion of the aberrant pool of HSPCs preceded clinical relapse (Craddock et al., 2013). A similar study of HSCs in lower-risk MDS has not been performed, but presumably MDS HSCs are relatively resistant to therapy, similar to that shown for lenalidomide.

Recently, the combination of AZA plus venetoclax, a BCL2 inhibitor, has been shown to be superior to AZA alone in several large clinical trials, inducing longer overall survival and higher incidence of remission in elderly AML and high-risk MDS patients (DiNardo et al., 2018; Ball et al., 2020; DiNardo et al., 2020; Pollyea et al., 2021). Analyzing LSCs from patients undergoing treatment with venetoclax plus AZA, Pollyea et al. showed that combining venetoclax and AZA eradicates LSC, at least in part, by disruption of the tricarboxylic acid (TCA) cycle and inhibition of electron transport chain complex II (Pollyea et al., 2018).

### MDS-SC Antigens and Novel Therapeutics

The ability to distinguish MDS-SCs from normal or preleukemic HSPCs in MDS patients would not only allow more rigorous investigations of MDS-SCs, but also identify potential MDS-SC therapeutic targets. Recent studies have identified surface antigens that are upregulated on MDS-SCs. In lower-risk MDS, the number of cell surface markers shown to be upregulated on HSCs is limited. We demonstrated that CD99/MIC2 is frequently overexpressed in MDS HSCs as well as AML LSC compared to their normal hematopoietic counterparts (Corces-Zimmerman et al., 2014; Chung et al., 2017). In addition, monoclonal antibodies (mAbs) targeting CD99 can induce cell death of MDS HSPCs and AML *in vitro*, and exhibit antileukemic activity in AML xenografts, without significantly depleting normal HSCs. CD99 may promote MDS-SC and AML LSC self-renewal ability by activating downstream pathways like SRC family kinases (Corces-Zimmerman et al., 2014; Chung et al., 2017). Although the precise functions and mechanisms of action of CD99 in MDS and AML remain to be determined, these studies demonstrate that CD99 is an attractive candidate for targeting stem cells in lower-risk MDS as well as AML.

### Surface Markers in High-Risk MDS/AML

Several surface antigens have been proposed as MDS-therapeutic targets, primarily in the context of high-risk MDS/AML.

IL1RAP (IL1R3) is overexpressed on HSPCs in high-risk MDS, but not in low-risk MDS. IL1RAP expression is positively correlated with overall survival in MDS and AML (Barreyro et al., 2012), and antibodies targeting IL1RAP showed therapeutic efficacy in xenograft models of AML (Askmyr et al., 2013; Ågerstam et al., 2015). Mechanistic studies have shown that IL1RAP function is not restricted to the IL-1 receptor pathway, but also mediates signaling and pro-proliferative effects through FLT3 and c-KIT signaling in AML LSCs (Mitchell et al., 2018).

High levels of CD123 (IL3-R alpha chain) expression in immature HSPCs (CD34+/CD38−) has been reported in high-risk, but not lower-risk MDS (Xie et al., 2010; Li et al., 2014). CD123 is also used to distinguish AML LSCs from normal HSCs and thus represent a desirable therapeutic target in AML (Jordan et al., 2000; Jin et al., 2009). CD123+ MDS-SCs also appear to exhibit a unique metabolic signature enriched in oxidative phosphorylation that is associated with stem cell self-renewal and survival in high-risk MDS (Stevens et al., 2018). SL-401, a diphtheria toxin interleukin-3 fusion protein, has been shown to induce cytotoxicity in CD123+ blasts from high-risk MDS and AML patients, and a phase 1/2 clinical trial of SL-401 as single agent therapy showed a predictable and manageable safety profile in patients with myeloid malignancies including high-risk MDS, CMML and blastic plasmacytoid dendritic cell neoplasm (BPDCN) (Alkharabsheh and Frankel, 2019). Another CD123 conjugate, IMGN632, is being evaluated as a single agent therapy as well as in combination with venetoclax and AZA in relapsed/refractory AML (Table 1). CD123-specific chimeric antigen receptor (CAR)-T cells have been developed and have been shown to eliminate LSCs in AML xenografts (Mardiros et al., 2013). MB-102, a CAR T cell therapy, is reported to induce complete responses at low doses in AML and BPDCN without dose-limiting toxicities in a phase I clinical trial (Table 1) (Budde et al., 2017). Although IMGN632 and MB-102 showed promising results in AML and BPDCN, the efficacy of IMGN632 and MB-102 in high-risk MDS needs to be evaluated in future clinical trials.

**TABLE 1:** Therapies targeting MDS-SCs in clinical trials.

Target	Disease Association	Drug name	Drug type	Intervention	Disease treated	Trial phase identifier
CD123	Overexpressed in HR MDS and AML	SL-401	ADC	SL-401 +	HR MDS and AML	Phase I/II: NCT03113643, NCT00397579
				HMAs +		
				Venetoclax		
		IMGN632	ADC	IMGN632	MPN, AML, BPDC, ALL	Phase I/II: NCT03386513
		MB-102	CAR-T	IMGN632 +	MPN, AML, BPDCN,ALL	Phase I: NCT02159495
				Cyclophosphamide +		
				Fludarabine + Phosphate		
TIM3	Overexpressed in HR MDS and AML	MBG453	Blocking antibody	MBG453 +	HR MDS and AML	Phase III: NCT04266301, Phase II: NCT03946670
				HMAs		
CD47	Overexpressed in HR MDS and AML	Magrolimab	Blocking antibody	Magrolimab +	HR MDS and AML	Phase III: NCT04313881
				Azacitidine		Phase I: NCT03248479
TLR2	Overexpressed in LR MDS	Tomaralimab	Blocking antibody	Tomaralimab	LR MDS	Phase I/II: NCT02363491, NCT03337451
CXCR2	Overexpressed in MDS	SX-682	Small molecular inhibitor	SX-682	HR MDS	Phase I: NCT04245397
IRAK1	Overexpressed in MDS	Pacritinib	Small molecular inhibitor	Pacritinib +	LR MDS	Phase I: NCT02469415, NCT02564536
				Azacitidine		
IRAK4	Overexpressed in MDS	CA-4948	Small molecular inhibitor	CA-4948	HR MDS and AML	Phase I: NCT04278768
STAT3	Overexpressed in MDS	Pyrimethamine	Small molecular inhibitor	Pyrimethamine	HR and IR MDS	Phase I: NCT03057990
SF3B1	Frequently mutated in MDS	H3B-8800	Small molecular inhibitor	H3B-8800	MDS and AML	Phase I: NCT02841540

Abbreviations: MDS, myelodysplastic syndrome; AML, acute myeloid leukemia; BPDCN, Blastic plasmacytoid dendritic cell neoplasm; MPN, myeloproliferative neoplasm; ALL, acute lymphoblastic leukemia; ADC, Antibody-drug conjugate; CAR-T, Chimeric antigen receptor T cell therapy; HR, High-risk; LR, Low-risk.

CD47 is an antiphagocytic marker that is overexpressed in many human cancers, allowing them to evade immunosurveillance by providing a “don’t eat me” signal (Pang et al., 2013). CD47 is significantly upregulated on committed myeloid progenitors in MDS, which we proposed protects MDS HSPCs from phagocytosis (Pang et al., 2013; Jiang et al., 2013). Thus, CD47 may serve as an important biomarker portending the transition from lower-risk MDS to high-risk MDS (Pang et al., 2013). Magrolimab (previously named 5F9) is a first-in-class antibody that blocks CD47, induces tumor phagocytosis, and eliminates LSCs in AML models (Sallman et al., 2019). AZA synergizes with magrolimab by inducing "eat me" signals to enhance phagocytosis (Sallman et al., 2019). A phase 1b clinical trial of magrolimab plus AZA in high-risk MDS/AML patients demonstrated that this combination shows an excellent safety profile with high response rates in both diseases (Sallman et al., 2019). Registrational clinical trial studies in expansion patient cohorts with high-risk MDS are ongoing (Table 1). Whether or not CD47-directed therapies will show efficacy in the context of low-risk MDS remains uncertain since CD47 upregulation on HSPCs was shown to occur primarily in cases with excess blasts (Pang et al., 2013).

T-cell immunoglobulin mucin-3 (TIM3) is overexpressed on LSCs in AML compared to normal HSPCs. TIM-3 and its ligand, galectin-9 (Gal-9), form an autocrine loop critical for AML LSC self-renewal (Kikushige et al., 2015). In MDS, TIM-3 and Gal-9 have been reported to be overexpressed in high-risk MDS and associated with worse clinical outcomes (Asayama et al., 2017). MBG453, a high-affinity humanized anti-TIM-3 IgG4 antibody, blocks TIM-3 function and is currently being evaluated in clinical trials of patients with high-risk MDS and AML (Table 1) (Borate et al., 2019; Zeidan et al., 2019).

As discussed above, many studies have demonstrated activation of inflammatory and innate immunity pathways in MDS-SCs, and targeting these pathways has shown to be beneficial in preclinical models. Innate immune sensors such as TLR2 are consistently overexpressed in MDS HSPCs from low-risk MDS patients, and high expression of TLR2 is correlated with disease progression. Tomaralimab (OPN-305), an antagonistic IgG4 mAb targeting TLR2, induces differentiation of erythroid cultures (Garcia-Manero et al., 2018). A phase 1 clinical trial of Tomaralimab in low risk/Int-1 MDS patients who previously failed HMA treatment showed good efficacy and safety (Garcia-Manero et al., 2018; Reilly et al., 2013). CXCR2 is an innate immune sensor that closely interacts with IL8 in mediating the activation of innate immunity pathways in malignant stem cells in MDS and AML (Schinke et al., 2015). Several small-molecule antagonists of CXCR2 are currently in development (Table 1) (Ghobrial et al., 2019; Bockorny et al., 2020). Given that IRAK1 and IRAK4 mediate chronic inflammatory signals in MDS, autoimmune disease, and other diseases such as MPNs and diffuse large B-cell lymphoma, there is great interest in IRAKs as therapeutic targets, and several IRAK inhibitors are currently under investigation in early clinical trials (Table 1) (Wiese et al., 2020; Rosenthal et al., 2019). Indeed, a combination of IRAK1 and BCL2 inhibitors has been shown to effectively eliminate MDS clones in xenografts (Rhyasen et al., 2013). Pacritinib is a small molecule that binds to the IRAK1 kinase domain with high-affinity and blocks IRAK1 phosphorylation (Hosseini et al., 2018). Pacritinib reduced AML cell growth *in vitro* and leukemia burden in AML xenografts (Jeon et al., 2020). IRAK4 inhibitors also are also being investigated in preclinical models (Wiese et al., 2020). Efforts to develop small-molecule inhibitors for other kinases and regulators including PAK1 and STAT3 are also in progress (Table 1) (Semenova and Chernoff, 2017; Yang et al., 2019).

Recent studies have demonstrated that MDS and AML cells bearing mutations in RNA spliceosome components including SF3B1, SRSF2 and U2AF1 are preferentially sensitive to spliceosome inhibition (Fei et al., 2016; Lee et al., 2016; Obeng et al., 2016; Shirai et al., 2017). These findings are presumed to be due to the fact that these cells only possess one functionally intact spliceosome gene allele, and therefore cannot tolerate further reductions in spliceosome function. Small molecule inhibitors including sudemycin, E7107 and H3B-8800 have been developed to target spliceosomal components, and although they are not selective for mutant spliceosome components, have been shown to reduce disease burden in preclinical AML and MDS mouse models (Lee et al., 2016; Shirai et al., 2017; Seiler et al., 2018). E7107, a derivative of the natural product pladienolide B, was investigated in a phase 1 clinical trial in advanced solid tumors, but the clinical trial was discontinued prematurely due to vision loss, which was reported as a major adverse event (Hong et al., 2014). H3B-8800, an orally available small-molecule inhibitor targeting SF3B1, selectively eradicates SF3B1-mutant leukemia cells in xenografts (Seiler et al., 2018) and is currently been investigated in a phase 1/2 clinical trial in AML and MDS (Table 1).

## Conclusion

Our understanding of the role of disease-initiating stem cells in MDS has improved tremendously in the past decade. The identification of recurrent somatic mutations in MDS has enabled numerous investigations of specific genes in hematopoiesis, while the evaluation of highly purified MDS-SCs, both in bulk and at the single cell level, has revealed insights into the genetic composition, order of mutational acquisition, mechanisms of disease progression, and mechanisms determining responses to therapy. Improved xenograft models have allowed investigators to interrogate MDS-SC function using patient cells rather than rely solely on mouse or cell line models. Collectively, these studies have demonstrated that dysregulated gene regulation, increased inflammatory signaling, alterations in RNA splicing, and alterations in ribosome assembly/translation are major drivers of MDS HSC function. They also identified cell surface markers including CD123, CD47, TIM3, and CD99 as potential therapeutic targets on MDS-SCs. While much work is still required to develop therapies that can deplete MDS-SCs and induce durable remissions or even cures, there is much reason to be hopeful that such strategies will emerge in the near future. Given the importance of understanding MDS-SC responses to these therapies, we advocate that studies of MDS-SC responses to therapy be performed in conjunction with clinical trials in order to provide the molecular insights necessary to design the next generation of combinatorial therapies. We also encourage investigators to design future studies to distinguish between molecular mechanisms required for SC function in high-risk vs. low-risk MDS, as well as between the types of clinical responses observed (e.g., hematologic improvement vs. blast reductions), in order to better understand the molecular bases of these distinct disease processes.
